# A functional variant in ST2 gene is associated with risk of hypertension *via* interfering MiR‐202‐3p

**DOI:** 10.1111/jcmm.13058

**Published:** 2017-01-25

**Authors:** Fangqin Wu, Lu Li, Qiang Wen, Jinhua Yang, Zhuyue Chen, Peng Wu, Meian He, Xiaomin Zhang, Tangchun Wu, Longxian Cheng

**Affiliations:** ^1^ Department of Cardiovascular MedicineThe Second Affiliated Hospital of Nanchang UniversityNanchangJiangxiChina; ^2^ Department of CardiologyUnion HospitalTongji Medical CollegeHuazhong University of Science and TechnologyWuhanHubeiChina; ^3^ Second Affiliated HospitalShantou University Medical CollegeShantouChina; ^4^ Institute of Occupational Medicine and the Ministry of Education Key Lab of Environment and HealthSchool of Public HealthTongji Medical CollegeHuazhong University of Science and TechnologyWuhanHubeiChina

**Keywords:** ST2, miR‐202‐3p, functional polymorphism, essential hypertension

## Abstract

Recent studies have suggested that interleukin 1 receptor‐like 1 (ST2) plays a critical role in pathogenesis of several cardiovascular disease conditions. In this study, we examined association of 13 single nucleotide polymorphisms (SNPs) of ST2 gene with essential hypertension (EH) risk in 1151 patients with EH and 1135 controls. Our study showed that variants rs11685424, rs12999364 and rs3821204 are highly associated with an increase in risk of EH, while rs6543116 is associated with a decrease risk of EH. Notably, in silico analyses suggested the G>C change of rs3821204, which located within the 3′UTR of soluble *ST2* mRNA, disrupted a putative binding site for miR202‐3p. Functional analyses suggested that miR‐202‐3p significantly decreased soluble ST2‐G mRNA stability and inhibited its endogenous expression. Furthermore, we found increased plasma‐soluble ST2 (sST2) level was highly associated with *CC* genotype of rs3821204 *in vivo*. Taken together, our findings provide the first evidence that genetic variants in *ST2* gene are associated with EH risk and variant rs3821204 may influence the development of EH by controlling sST2 expression.

## Introduction

Hypertension is a significant public health problem worldwide and affects more than 30% of the population 1. EH is multifactorial disease for which genetic and environmental factors (such as diet rich in sodium, alcohol and smoking) are determinants 2, 3. Thus, identification of susceptibility genes for EH provides insights into the disease pathogenesis 4, 5.

It is well known that the interleukin 33 (IL‐33)/interleukin 1 receptor‐like 1 (ST2) pathway plays an important role in pathogenesis of cardiovascular disease such as atherosclerosis, heart failure and hypertension 6, 7. The *ST2* gene is located on chromosome 2q12 and contains 11 exons, a proximal promoter and a distal promoter. This gene encodes an IL‐33 receptor which consists of two main isoforms, a transmembrane ligand (ST2L) and a soluble component (sST2, soluble ST2) 8. IL‐33 and ST2L form a heterodimeric receptor complex with IL‐1R accessory protein (IL‐1RAcP), triggering selective immune and inflammatory responses that result in antihypertrophic and antifibrotic myocardial effects 9, 10, 11, 12. Meanwhile, sST2 binds to IL‐33, and functions as a decoy receptor for IL‐33 signal, resulting in inhibition of cardiovascular disease benefits 11, 13, 14. Clinically, sST2 is associated with hypertension, and increased sST2 levels predict increased systolic blood pressure (SBP) 7, 15. Moreover, sST2 is reported as a novel cardiac biomarker of mechanical strain, which was recently shown to be increased in hypertension 16, 17.

Although higher sST2 concentration has been reported in association with hypertension, association between *ST2* polymorphisms and EH has not been systematically investigated. Accordingly, we suggested that genetic variations of the *ST2* gene influence EH development by interfering the function or expression of ST2. To investigate this, we performed a genetic association analysis of *ST2* and EH risk in a case–control study of the Han Chinese population. Further, we performed functional analyses to investigate the underlying functional role of *ST2* variants in EH development.

## Materials and methods

### Study population

The sample population included 1151 EH patients and 1135 controls. Patients were unrelated to each other and recruited from the Han Chinese population. EH patients were recruited from three hospitals (Union Hospital, Tongji Hospital and Wugang Hospital) in Wuhan City, Hubei province, China 18. Hypertension was diagnosed as average SBP ≥140 mmHg, and/or average diastolic pressure (DBP) ≥90 mmHg, and/or self‐reported current treatment for hypertension with antihypertensive drugs. Control subjects were from the same community as the patients. Control subjects showed SBP <140 mmHg, DBP <90 mmHg and had never been treated for hypertension. Blood pressure was measured according to reported guidelines 2. All patients completed an Inter‐Heart questionnaire and were interviewed by trained interviewers for their demographic data, medical history, history of disease, family history of cardiovascular disease and lifestyle habits (including smoking and alcohol consumption). Six normal tissues (heart, kidney, liver, peripheral blood mononuclear cells (PBMCs), spleen and vein) were obtained at Union Hospital, and immediately stored in liquid nitrogen after surgery until further use. Each tissue was from four different donors. The study was approved by the Ethics Committee of Tongji Medical College, and all participants provided written informed consent and adhered to the principles of the Declaration of Helsinki.

### Selection of polymorphisms

TagSNPs were selected based on the HapMap phase I & II database [http://www.hapmap.org; with CHB (Han Chinese in Beijing, China) and JPT (Japanese in Tokyo, Japan) as the reference set]. According to the criteria of *r* ^2^ ≥ 0.8 and minor allele frequency (MAF) ≥0.05, to cover the distal promoter region of *ST2*, we extended 30 kb into the 5′ and 2000 bp into the 3′ end of *ST2*, and selected six tagSNPs to be analysed. Another seven SNPs were selected from previous reports of coronary heart disease (CHD) (‐27307T/A and ‐27614C/A), asthma and atopic dermatitis 19, 20, 21. In total, 13 SNPs were selected and genotyped (Table 1).

**Table 1:** SNP locations and allele frequencies

SNP	Location	Genotype	MAF^a^	HWE *P* a
rs3755278	Intron	A/G	0.067	1
rs10206753	Exon	C/T	0.133	1
rs1041973	Exon	A/C	0.156	0.527
rs11685424	Distal promoter	A/G	0.487	0.05
rs6543116	Distal promoter	A/G	0.367	–
rs951774	Distal promoter	A/C	0.233	0.251
rs10515922	Distal promoter	C/T	0.151	1
rs13006559	Distal promoter	T/C	0.07	1
‐27307T/A	Distal promoter	T/A	–	–
‐27614C/A	Distal promoter	C/A	–	–
rs12999364	Between genes	T/C	0.45	0
rs3821204	3′‐Flanking region	C/G	0.358	0.403
rs13431828	5′‐Flanking region	T/C	0.089	1

a National Center for Biotechnology Information (NCBI) data; –, data not found in http://www.ncbi.nlm.nih.gov/snp/.

### Bioinformatics

Appropriate bioinformatics websites were used to predict the putative binding site for rs3821204: http://snpinfo.niehs.nih.gov/snpinfo/snp func.htm and http://epicenter.ie-freiburg.mpg.de/services/microsniper/.

### DNA isolation and genotyping

Fasting venous blood was collected from the peripheral vein. DNA was extracted using a Puregene kit (Gentra Systems, Inc., Minneapolis, MN, USA). Twelve SNPs were genotyped using the Sequenom MassArray system (Sequenom Inc., San Diego, CA, USA). The primers and probes used are listed in Table S1. The remaining SNP, rs3755278, was genotyped by TaqMan SNP allelic discrimination (Applied Biosystems, Foster City, CA, USA). The catalogue number for genotyping rs3755278 was C_27477453_10. TaqMan data collection and analysis were performed with SDS 2.2.1. Overall, all 13 SNPs were successfully genotyped with a call rate >96%. About 10% of the samples were randomly selected for repeat genotyping of the three SNPs for quality control, and the results were 100% concordant.

### Cell culture and genotyping

The human umbilical vein cell line, EA.hy926, human embryonic kidney cell line, HEK293, and T‐acute lymphoblastic leukaemia cell line, KOPTK1, were obtained from American Type Culture Collection (ATCC) and cultured according to the manufacturer's protocols.

Genomic DNA was isolated from cells using the Guide Cell and Tissue Genomic DNA Extraction Kit (Tiangen, Beijing, China). The genotypes of cell lines were determined by Taqman genotyping assay.

### Biological variable determination

Total cholesterol (TC), triglyceride (TG) and fasting blood glucose (FBG) levels were measured by standard laboratory procedures at the Department of Clinical Laboratory, Union Hospital.

### RNA isolation and reverse transcription PCR

Total RNA was extracted using Trizol (Life Technologies, Carlsbad, CA, USA). For reverse transcription PCR (RT‐PCR), cDNA was obtained using the SuperScriptIII^**®**^ Frist‐Strand Synthesis System (Life Technologies). Gene expression was monitored by PCR assay. The primer sequences were as follows: sST2 forward, 5′‐ GGCACACCGTAAGACTAAGTAG‐3′ and sST2 reverse, 5′‐CAATTTAAGCAGCAGAGAAGCTCC‐3′; β‐actin forward, 5′‐CCCAGCCATGTACGTTGCTAT‐3′ and β‐actin reverse, 5′‐TCACCGGAGTCCATCACGAT ‐3′. Levels of miR‐202‐3p and U6 were monitored by quantitative RT‐PCR using the Bulge‐Loop™ miRNA RT‐PCR primer set (RiboBio Co., Ltd. Guangzhou, China).

### UTR cloning and point mutant construction

The 3′‐untranslated region (UTR) fragment of *ST2* was amplified with primers: forward, 5′‐GTCTCTAGAATCCCCCACTCCCTCC‐3′ and reverse, 5′‐CAGTCTAGAATCTGTGTTCCTGCCC‐3′. A point mutation (G>C) was introduced by recombinant PCR using the primers: forward, 5′‐GTTTTTCTGGTCATAATGAAC‐3′ and reverse, 5′‐GTGTTCATTATGACCAGAAAAACGTATAGAACGG‐3′. Fragments were digested with *Xba*I and inserted into the luciferase reporter vector, pGL3‐control (Promega, Madison, WI, USA).

### Dual‐luciferase reporter gene assay

HEK293 cells were seeded into 24‐well plates at 20,000 cells/well, and incubated at 37°C for 24 hrs. Luciferase constructs (with ~1‐kb sST2 3′‐UTR mRNA) and micro RNA (miRNA) mimics were cotransfected using lipofectamine 2000 (Life Technologies), following the manufacturer's instructions. At 48 hrs after transfection, cells were lysed and luciferase assays performed with the Dual‐Luciferase Reporter Assay System (Promega) on a single automatic injection Mithras luminometer (Berthold Technologies, Bad Wildbad, Germany), following the manufacturer's instructions. Ratios of firefly luciferase to *Renilla* luciferase readings were calculated.

### Plasma sST2 levels determination

Fifty participants were selected from the control group. Plasma sST2 concentrations were measured using a highly sensitive sandwich immunoassay: Pressage ST2 (Critical Diagnostics, San Diego, CA, USA). Test procedures were performed according to the manufacturer's instructions. The lower limit of sST2 detection was 3.1 ng/ml and the upper limit 200 ng/ml.

### Statistical analysis

Continuous variables were expressed as mean ± standard deviation (S.D.). Distribution of numerical data was determined by the Kolmogorov–Smirnov normality test. Continuous data with a normal distribution were compared by Student's *t*‐test and those with unequal variance or without a normal distribution were analysed by Mann–Whitney rank‐sum tests. Chi‐square tests were used to compare categorical variables and Hardy–Weinberg equilibrium (HWE) of SNPs. Unconditional logistic regression analysis was used to determine association between SNPs and hypertension risk, after adjustment for age, sex, smoking, drinking, body mass index (BMI), TG, FBG and family history of hypertension by odds ratios (ORs) and 95% confidence intervals (CIs). Power calculations were performed with the QUANTO software program (Version 1.2.3) (University of Southern California, Los Angeles, CA, USA). One‐way analysis of variance (anova) was used to examine differences in sST2 levels in patients with different rs3821204 genotypes. All other statistical analyses were performed with the statistical analysis software package SPSS 12.0 (SPSS Inc., Chicago, IL, USA). Differences or associations with two‐sided *P*‐values <0.05 were considered statistically significant.

## Results

### Characteristics of case and control patients

General participant characteristics are presented in Table 2. There were a total of 2286 participants, including 1151 EH patients and 1135 controls. Male and female numbers in both groups were similar. However, EH patients were older and had greater BMI and higher SBP, DBP, FBG and TG levels than controls. Further, EH patients were more likely to have a history of CHD, diabetes mellitus and a family history of hypertension. TC levels were significantly lower in EH patients than controls, possibly due to use of cholesterol‐lowering medications in the patient group.

**Table 2:** General characteristics of the study population

Variables	Controls (*n* = 1135)	Cases (*n* = 1151)	*P* value
Sex, m/f (%)	888/247(78.2/21.8)	899/252(78.1/21.9)	0.939^a^
Age, years	58.2 ± 11.7	62.3 ± 9.5	<0.01^b^
Blood pressure, mmHg			
Systolic	125.5 ± 27.0	143.8 ± 24.2	<0.01^b^
Diastolic	78.4 ± 11.3	86.3 ± 14.1	<0.01^b^
Body mass index, kg/m^2^	23.4 ± 3.2	24.7 ± 3.2	<0.01^b^
Fasting glucose, mmol/l	5.6 ± 2.2	6.2 ± 3.0	<0.01^b^
Total cholesterol, mmol/l	4.59 ± 1.00	4.44 ± 1.04	<0.01^b^
Triglyceride, mmol/l	1.57 ± 1.26	1.72 ± 1.41	<0.01^b^
Smoking, no/yes (%)	660/475(58.1/41.9)	796/353(69.3/30.7)	<0.01^a^
Drinking, no/yes (%)	766/365(67.7/32.3)	830/314(72.6/27.4)	0.012^a^
Past history			
Coronary heart disease, no/yes	784/351(69.1/30.9)	361/790(31.4/68.6)	<0.01^a^
Diabetic patient, no/yes	1041/92(91.9/8.1)	861/287(75.0/25.0)	<0.01^a^
Family history of Hypertension, no/yes (%)	862/266(76.4/23.6)	689/443(60.9/39.1)	<0.01^a^

Variables are presented as mean ± S.D. or percentages.

a *P*‐values were calculated using chi‐square tests.

b *P*‐values were calculated using independent‐samples *t*‐tests.

### Association between *ST2* variants and EH risk

Most of the SNPs conformed to HWE (*P* > 0.05), except for rs1041973 and rs10515922, which significantly deviated from HWE in EH patients and controls (*P* < 0.05) (see Table S2). MAFs for ‐27307T/A and ‐27614C/A were 0, and according to the HapMap database, these alleles have not previously been identified in the Chinese Han population. Consequently, we examined association between the remaining nine SNPs and EH risk. To determine association between these SNPs and EH risk, we analysed genotypes using additive and recessive models. Four of these SNPs (rs11685424, rs6543116, rs12999364 and rs3821204) were significantly associated with EH after adjusting for conventional EH risk factors such as age, sex, smoking, drinking, BMI, TG, FBG and family history of hypertension (Table 3).

**Table 3:** Genotype frequencies of nine SNPs and their association with essential hypertension risk in the Chinese population

SNPs	Controls^b^	Cases^b^	Additive model^c^		Recessive model^d^	
			OR (95% CI)^a^	*P* a	OR (95% CI)^a^	*P* a
rs3755278	1021/76/4	994/99/3	1.34(0.94–1.90)	0.102	1.40(0.97–2.02)	0.071
rs10206753	826/257/29	841/266/20	0.91(0.75–1.11)	0.339	0.93(0.75–1.17)	0.536
rs11685424	307/537/247	253/581/282	**1.25(1.09**–**1.44)**	**0.002**	**1.33(1.06**–**1.66)**	**0.015**
rs6543116	312/542/252	350/566/207	**0.82(0.71**–**0.94)**	**0.005**	**0.74(0.60**–**0.92)**	**0.007**
rs951774	626/408/73	661/411/55	0.92(0.78–1.08)	0.312	0.95(0.78–1.16)	0.592
rs13006559	1052/57/3	1051/79/2	1.25(0.84–1.88)	0.278	1.36(0.89–2.08)	0.158
rs12999364	437/502/169	376/560/190	**1.26(1.09**–**1.45)**	**0.002**	**1.40(1.14**–**1.71)**	**0.001**
rs3821204	478/493/134	428/540/157	**1.25(1.08**–**1.45)**	**0.003**	**1.33(1.09**–**1.63)**	**0.005**
rs13431828	921/177/11	933/183/13	0.98(0.77–1.23)	0.845	0.97(0.75–1.26)	0.827

It means *P*‐values <0.05 and these SNPs were significantly associated with EH.

a *P*‐values were calculated by unconditional logistic regression, after adjusting for age, sex, smoking, drinking, BMI, TG, FBG and family history of EH.

b Wild‐type homozygote/heterozygote/variant homozygote.

c Additive model (wild‐type homozygote *versus* heterozygote *versus* variant homozygote).

d Recessive model (wild‐type homozygote *versus* heterozygote + variant homozygote).

Specifically, we found that the A allele of rs11685424 was associated with increased EH risk in both additive (*P* = 0.002, OR = 1.25, 95% CI = 1.09–1.44) and recessive (*P* = 0.015, OR = 1.33, 95% CI = 1.06–1.66) models. In contrast, the rs6543116 A allele was associated with decreased EH risk in both additive (*P* = 0.005, OR = 0.82, 95% CI = 0.71–0.94) and recessive (*P* = 0.007, OR = 0.74, 95% CI = 0.60–0.92) models. Additionally, there was a dose–response effect for increasing number of rs6543116 A alleles and decreased EH risk. Both rs12999364 and rs3821204 were also associated with EH risk in both additive (*P* = 0.002, OR = 1.26, 95% CI = 1.09–1.45 and *P* = 0.003, OR = 1.25, 95% CI = 1.08–1.45, respectively) and recessive (*P* = 0.001, OR = 1.40, 95% CI = 1.14–1.71 and *P* = 0.005, OR = 1.33, 95% CI = 1.09–1.63, respectively) models. Overall, our findings suggest increased EH risk in individuals carrying these homozygous *ST2* variants. Except for rs11685424 and rs6543116 in the recessive model, *P*‐values for these SNPs reached significance after Bonferroni correction (*P* < 0.0055; 0.05/9). There were no significant associations between the remaining five SNPs and EH risk in our population (*P* > 0.05). Given the frequency of CHD within this case–control sample population, we analysed subgroups without CHD patients to determine whether the effect was influenced by CHD risk. Exclusion of individuals with CHD did not change the results (see Table S3).

### The *ST2* polymorphism rs3821204 affects sST2 mRNA stability

**Figure 1:**
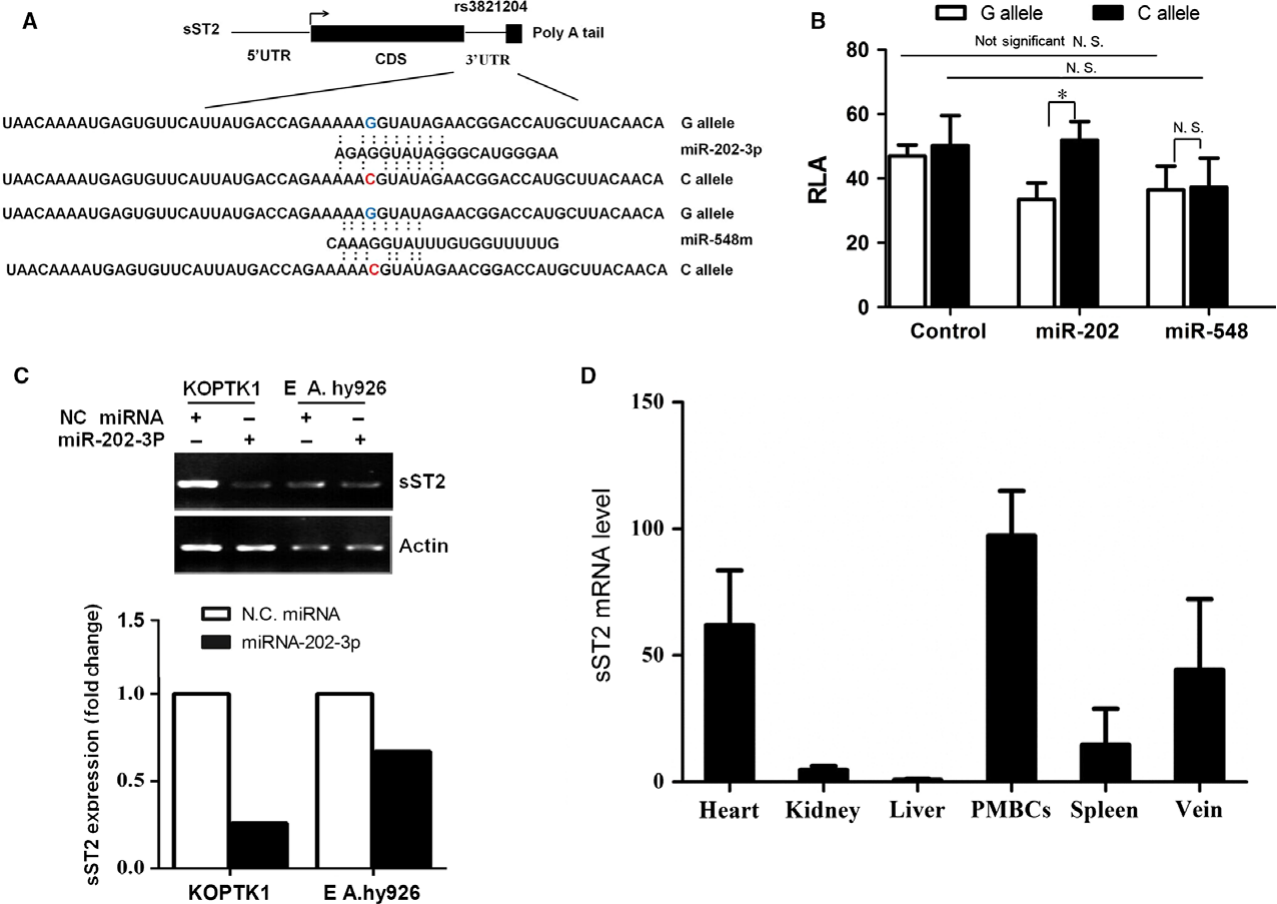
Allelic difference of rs3821204 G>C in sST2 mRNA stability and expression (**A**) In silico prediction of the miR‐202‐3p binding site in sST2‐G mRNA. Putative rs3821204 function was determined using the SNPinfo database. (**B**) The effect of miR‐202‐3p using RLA of sST2‐3′‐UTR fusion constructs encompassing different alleles. Constructs containing G or C rs3821204 alleles were cotransfected with miR‐202‐3p or a negative control. Relative luciferase activity was analysed at 48 hrs post‐transfection. Data represent mean ± S.D. from three independent experiments. *P < 0.05. (**C**) Allelic difference in endogenous sST2 mRNA levels after miRNA‐202‐3p transfection. Genotypes of rs3821204 were determined by Taqman assay in KOPTK1 and EA.hy926 cells. miRNA‐202‐3p mimics were transfected into KOPTK1 (GG) and EA.hy926 (CC) cells. At 48 hrs post‐transfection, cells were harvested to isolate total RNA. sST2 mRNA levels were monitored to determine the effect of miR‐202‐3p on sST2‐G and sST2‐C degradation. (**D**) miR‐202‐3p expression in different human tissues. Total RNA was isolated from six tissues from four different individuals. miRNA levels were determined by quantitative RT‐PCR using a Bulge‐Loop™ miRNA RT‐PCR primer set. U6 was used as the loading control. miR‐202‐3p in different tissues was expressed relative to liver miR‐202‐3p expression.

In silico analysis showed that the variants, rs11685424, rs12999364, and rs6543116, disrupted transcription factor binding sites, while rs3821204 disrupted a miRNA seeding site (Table S4). Together, this suggests these variants may influence EH susceptibility by controlling their own expression. Previous studies have confirmed this, and shown that rs11685424 and rs6543116 are associated with elevated EH risk and increased sST2 expression 19, 22. As noted, rs3821204 disrupts a putative miRNA binding site; therefore we predicted that this variant controls *ST2* expression *via* a mechanism that is distinct compared with the three other SNPs. To identify this mechanism, we performed a bioinformatics assay using the software, MicroSNiPer, to predict the putative function of rs3821204. We found that the G>C change associated with rs3821204 disrupts a putative binding site for miR‐202‐3p and miR‐548 m (Fig. 1A). Accordingly, we hypothesize that rs3821204 controls sST2 expression in a miRNA‐mediated post‐transcriptional manner.

As miRNAs often function as negative regulators of gene expression, we performed luciferase assays to determine the effect of rs3821204 on sST2 expression. We found that miR‐202‐3p significantly decreased RLA of ST2‐G but not ST2‐C, while miR‐548 m did not alter RLA of ST2‐G or ST2‐C (Fig. 1B). This is consistent with miR‐202‐3p overexpression, which caused a sharp reduction in endogenous sST2‐G mRNA levels, but not sST2‐C (Fig. 1C).

Additionally, we examined miR‐202‐3p tissue distribution by RT‐PCR. Greatest expression was observed in PBMCs, followed by the heart and vein (Fig. 1D). These findings are coincident with tissue distribution of sST2 protein, suggesting that regulation of sST2 by miR‐202‐3p might be critical to cardiovascular function.

### *In vivo* effect of the *ST2* polymorphism rs3821204 on plasma sST2 levels

**Figure 2:**
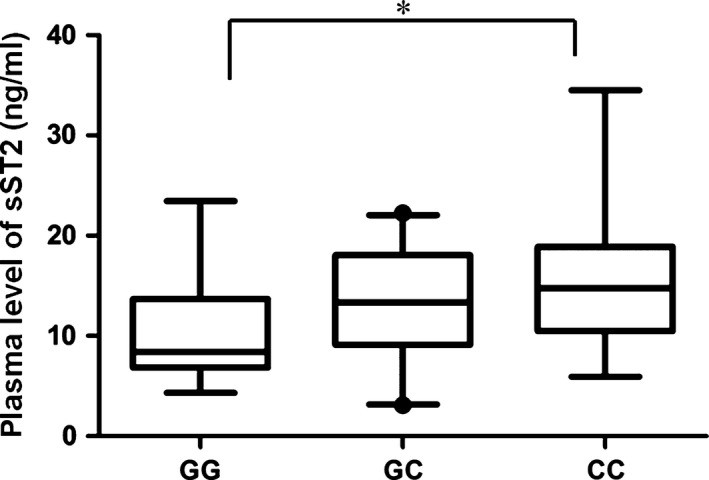
Circulating sST2 levels differ by rs3821204 genotype. Plasma concentrations of sST2 protein were measured by ELISA in 50 healthy donors carrying different rs3821204 genotypes (GG, *n* = 14; GC, *n* = 25; and CC, *n* = 11). Box plots indicate minimum, 10% percentile, maximum and 90% percentile for each group. *P < 0.05.

To examine the effect of rs3821204 on sST2 plasma levels, we performed ELISA and a Taqman genotyping assay in 50 healthy individuals (median [25–75th percentile], 13.28 ng/ml [8.03–16.91 ng/ml]). Plasma sST2 concentrations were significantly lower in patients with GG genotypes compared to those with CC genotypes (*P* < 0.05) (Fig. 2). These results suggest that rs3821204 G>C is associated with increased plasma sST2 levels *in vivo*.

## Discussion

In the present study, we have investigated association between 13 *ST2* gene polymorphisms and EH risk. Accordingly, we found four SNPs significantly associated with EH risk. Functional analyses showed the G>C change of rs3821204 disrupted a binding site for miR‐202‐3p and enhance sST2 mRNA expression by blocking miR‐202‐mediated degradation. In addition, patients with the rs3821204 C allele had significantly higher plasma sST2 levels. Taken together, our findings suggest that genetic variation within *ST2* modifies EH susceptibility by regulating its own expression.

The IL‐33/ST2 pathway plays an important role in inflammatory and immunity diseases, including asthma, atopic dermatitis and coronary artery disease 23. Recent research reported sST2 as a new prognostic biomarker in patients with hypertension 7, 15. Nonetheless, association between *ST2* polymorphisms and EH risk has not been systematically studied. In this case–control study, we found that the rs11685424 and rs6543116 genetic variations within the *ST2* promoter region are associated with EH risk. These two variants modify transcriptional activity of the ST2 promoter 19, 22. Thus, it is likely that the biological consequence of these genetic variants may influence EH susceptibility. Recently, Tu and Tsapaki reported association between *ST2* variants and CHD 20, 22. To minimize this disturbance, we re‐analysed association between *ST2* variants and EH risk in subgroups without CHD. Similar results were obtained, indicating that the four variants are associated with EH independent of CHD status. To the best of our knowledge, this is the first study to systematically investigate association between *ST2* genetic variants and EH risk, and provides obvious evidence for their association.

Micro RNAs are key regulators in the pathogenic processes of hypertension, and are involved in endothelial activation and dysfunction, vascular smooth muscle cell proliferation and inflammation 24, 25, 26, 27. Mounting evidence suggests that SNPs located within miRNA binding sites affect hypertension risk by interfering with miRNA function 28, 29, 30, 31. Our findings here demonstrate that the rs3821204 C allele disrupts the miR‐202‐3p binding site within the 3′‐UTR of sST2, and consequently disrupts sST2 mRNA expression and increases circulating sST2 protein levels (Figs. 1B and 2). Furthermore, our findings provide evidence of miR‐202‐3p expression in heart, PBMCs and vein, which is consistent with tissue distribution of sST 32, 33. Taken together, our results suggest that ST2 regulation by miR‐202‐3p is critical for maintaining normal cardiovascular activity, with its disruption having severe consequences.

Previous epidemiological studies have shown that elevated plasma sST2 concentration is associated with risk and outcome of hypertension 7, 15, 16, 17. Consistently, we also found that the rs3821204 C allele is associated with elevated plasma levels of ST2 (Fig. 2), and increased risk of EH (Table 3). Thus, our findings provide further evidence to support the important role of ST2 in EH development. However, the mechanism by which sST2 affects development of EH is poorly understood. Previous studies have shown that IL‐33 signalling *via* ST2L exhibits cardioprotective effects against various cardiovascular diseases (*e.g*. myocardial infarction, atherosclerosis, pulmonary arterial hypertension, cardiac hypertrophy and fibrosis) by modulating immune and inflammatory responses 6, 11, 34. Hence, we assume that sST2, an IL‐33 decoy receptor, may function in hypertension risk by disrupting IL33‐ST2L signalling.

In our present study, we had >90% power to detect association between the SNPs and EH risk, at a 0.05 significance level under both additive and recessive models, except for rs11685424, which had 84% power to detect association under the recessive model. Nevertheless, our study has several limitations. First, the study sample was not sufficiently large, especially when CHD patients were excluded. Further studies are needed to replicate our results in a larger sample size population. Second, our sample population was not well matched, but we addressed this using unconditional logistic regression analysis with adjustment for age, sex, smoking, drinking, BMI, TG, FBG and family history of hypertension. Third, all the volunteers recruited in our study were ethnic Han Chinese, and association between these SNPs and EH risk in other ethnic groups needs further validation in larger prospective studies.

In conclusion, we have found significant association between a *ST2* polymorphism and risk of EH. Additionally, our findings further implicate rs3821204 as a functional polymorphism that contributes to increase EH risk and enhanced sST2 expression by disruption of a miR‐202‐3p binding site. Thus, our findings imply that ST2 may strongly influence development of EH, further supporting the important role of ST2 in EH, and highlighting ST2 as a valuable target for prevention and treatment of EH.

## Conflict of interests

The authors confirm that there are no conflict of interests.

## Supporting information


**Table S1.** Primer sequences for genotyping 12 SNPs from the *ST2* gene using the Sequenom platform.

**Table S2.** Frequencies of 13 *ST2* SNPs in the study population.

**Table S3.** Secondary analyses of four SNPs significantly associated with EH risk in the subgroup without CHD subjects.

**Table S4.** Functional prediction of *ST2* variants.
