# Supplementary material for: A functional variant in ST2 gene is associated with risk of hypertension via interfering MiR‐202‐3p
Source: J Cell Mol Med. 2017 Jan 25;21(7):1292–9. doi: 10.1111/jcmm.13058 (PMC5487927; doi:10.1111/jcmm.13058)
Supplement: Supplementary file 1 — Table S1. Primer sequences for genotyping 12 SNPs from the ST2 gene using the Sequenom platform. [file JCMM-21-1292-s001.docx]

S1 Table. Primer sequences used for genotyping 12 SNPs on *ST2* gene with the Sequenom platform

| SNP | Forward primers(5'to 3') | Reverse primers(3'to 5') | Extension primers(5'to 3') |
| --- | --- | --- | --- |
| rs10206753 | ACGTTGGATGCATCACAGCAGGCACTATTG | ACGTTGGATGCCTGTGCCAAGCAAAATTCC | GGGGATATTGCTTCTGGGCAGCC |
| rs1041973 | ACGTTGGATGGGAGCCTTCTGCATAATACC | ACGTTGGATGAGGCCAACTTCTGAAGTTTC | TACCAGAATCAGCAACT |
| rs11685424 | ACGTTGGATGGAGCAGAAAATCACTAGTA | ACGTTGGATGCTCTCGTGTGAAGGGTATTG | ATCACTAGTATTTCATCAATATGGATA |
| rs6543116 | ACGTTGGATGCGCGTTAAGGGAAATGAGTG | ACGTTGGATGTTGCCTTCTTCATGAGTGAC | CTTGCCCTGCCACCTACATGGTGTT |
| rs951774 | ACGTTGGATGTTAGAAGTCTCTTGGGTGGG | ACGTTGGATGGCTCAGCCAGAGGTCTTTAC | AGGGGAAAAGGAAAGAT |
| rs10515922 | ACGTTGGATGGTTCTGTTCAAAAGTCAAGG | ACGTTGGATGAGGGCATTTGGTCTTGCATC | GTCAAGGATAGCATAAGACT |
| rs13006559 | ACGTTGGATGTTAGAATCTTCTCCCTCGCC | ACGTTGGATGGGGAGATGCTCATGTTATTG | TCCCTCGCCCCTAAC |
| -27307T/A | ACGTTGGATGGGGTTCTTGGCTTATGCAAC | ACGTTGGATGGAGCAAATTGGTCCACTTCC | CCCCAAGTGCTACTTACTAAGCC |
| -27614C/A | ACGTTGGATGGCACCTAAAGCTTTGGACAG | ACGTTGGATGTATTAACTGCTTGGCTGCTC | GGGTCCACGGAGAGGCCAAAGAT |
| rs12999364 | ACGTTGGATGATAGGCATGTGCCATTTGGG | ACGTTGGATGGTCCCTAGAGTCCTTTCAAC | CTTCCTTGAGAAGGCAAATA |
| rs3821204 | ACGTTGGATGACTTTACCACCCTCGCTAAC | ACGTTGGATGCATGTTGTAAGCATGGTCCG | GGATTGTTCATTATGACCAGAAAAA |
| rs13431828 | ACGTTGGATGAGAGTATCACCAACTGCCTC | ACGTTGGATGCGTTGTTGAGATTACTCCAG | CTAATGTGTGGTGACCTTCACTGT |
