# Supplementary material for: A functional variant in ST2 gene is associated with risk of hypertension via interfering MiR‐202‐3p
Source: J Cell Mol Med. 2017 Jan 25;21(7):1292–9. doi: 10.1111/jcmm.13058 (PMC5487927; doi:10.1111/jcmm.13058)
Supplement: Supplementary file 2 — Table S2. Frequencies of 13 ST2 SNPs in the study population. [file JCMM-21-1292-s002.docx]

**S2 Table. The frequencies of 13 *ST2* SNPs in the population under observation**

| SNP | Location | Genotype | MAF | HWE *P* |
| --- | --- | --- | --- | --- |
| rs3755278 | intron | A/G | 0.047 | 0.128 |
| rs10206753 | Exon | C/T | 0.139 | 0.293 |
| rs1041973 | Exon | A/C | 0.139 | < 0.001 |
| rs11685424 | Distal promoter | A/G | 0.493 | 0.531 |
| rs6543116 | Distal promoter | A/G | 0.454 | 0.907 |
| rs951774 | Distal promoter | A/C | 0.241 | 0.878 |
| rs10515922 | Distal promoter | C/T | 0.138 | 0.042 |
| rs13006559 | Distal promoter | T/C | 0.033 | 0.078 |
| -27307T/A | Distal promoter | T/A | 0 | 0 |
| -27614C/A | Distal promoter | C/A | 0 | 0 |
| rs12999364 | Between genes | T/C | 0.398 | 0.695 |
| rs3821204 | 3'-Flanking region | C/G | 0.362 | 0.898 |
| rs13431828 | 5'-Flanking region | T/C | 0.091 | 0.168 |
