# Supplementary material for: A functional variant in ST2 gene is associated with risk of hypertension via interfering MiR‐202‐3p
Source: J Cell Mol Med. 2017 Jan 25;21(7):1292–9. doi: 10.1111/jcmm.13058 (PMC5487927; doi:10.1111/jcmm.13058)
Supplement: Supplementary file 3 — Table S3. Secondary analyses of four SNPs significantly associated with EH risk in the subgroup without CHD subjects. [file JCMM-21-1292-s003.docx]

S3 Table. Secondary analyses of 4 SNPs significantly associated with EH risk in subgroup without CHD subjects

| SNPs | Case^a^ | Control^a^ | Additive Model ^b^ | | Recessive Model^c^ | |
| --- | --- | --- | --- | --- | --- | --- |
|  |  |  | OR (95% CI)^d^ | *P* ^d^ | OR (95% CI)^d^ | *P* ^d^ |
| rs11685424 | 73/185/88 | 224/360/172 | **1.42(1.13-1.77)** | **0.002** | **1.66 (1.15–2.39)** | **0.007** |
| rs6543116 | 105/183/58 | 220/354/187 | **0.72(0.57-0.90)** | **0.003** | **0.68 (0.49–0.96)** | **0.030** |
| rs3821204 | 124/181/41 | 328/340/93 | **1.36(1.08-1.72)** | **0.010** | **1.62 (1.17–2.24)** | **0.004** |
| rs12999364 | 107/178/61 | 300/348/113 | **1.43(1.14-1.79)** | **0.002** | **1.67 (1.20–2.33)** | **0.002** |

^a.^ Wild–type homozygote/heterozygote/variant homozygote.

^b.^ Additive Model (wild type homozygote vs. heterozygote vs. variant homozygote)

^c.^ Recessive Model (wild–type homozygote vs. heterozygote + variant homozygote)

^d.^ Data were calculated by unconditional logistic regression, adjusted for age, sex, smoking, drinking, BMI, TG, FBG, and family history of EH.
