# Supplementary material for: A functional variant in ST2 gene is associated with risk of hypertension via interfering MiR‐202‐3p
Source: J Cell Mol Med. 2017 Jan 25;21(7):1292–9. doi: 10.1111/jcmm.13058 (PMC5487927; doi:10.1111/jcmm.13058)
Supplement: Supplementary file 4 — Table S4. Functional prediction of ST2 variants. [file JCMM-21-1292-s004.docx]

**S4 Table. Function prediction of *ST2* variants**

| SNPs | Chrom-  osome | Position | Allele | TFBS | Splicing  (site) | Splicing  (ESE or ESS) | Splicing  (abolish domain) | miRNA  (miRanda) | nsSNP | Stop Codon | Polyphen | SNPs3D  (svm profile) | SNPs3D  (svm structure) |
| --- | --- | --- | --- | --- | --- | --- | --- | --- | --- | --- | --- | --- | --- |
| rs11685424 | 2 | 102293413 | A/G | Y | -- | -- | -- | -- | -- | -- | -- | -- | -- |
| rs12999364 | 2 | 102340561 | C/T | Y | -- | -- | -- | -- | -- | -- | -- | -- | -- |
| rs3821204 | 2 | 102326713 | G/C | -- | -- | -- | -- | Y | -- | -- | -- | -- | -- |
| rs6543116 | 2 | 102294158 | A/G | Y | -- | -- | -- | -- | -- | -- | -- | -- | -- |

***** The assay was conducted with SNPinfo Web Server.
